# Changes in mental distress among employees during the three years of the COVID-19 pandemic in Germany

**DOI:** 10.1371/journal.pone.0302020

**Published:** 2024-05-03

**Authors:** Swaantje Casjens, Dirk Taeger, Thomas Brüning, Thomas Behrens

**Affiliations:** Institute for Prevention and Occupational Medicine of the German Social Accident Insurance, Institute of the Ruhr University Bochum (IPA), Bochum, Germany; University of Pretoria, SOUTH AFRICA

## Abstract

**Objectives:**

The COVID-19 pandemic changed the future of work sustainably and led to a general increase in mental stress. A study conducted during the second and third pandemic wave with a retrospective survey of the first wave among 1,545 non-healthcare workers confirmed an increase in anxiety and depression symptoms and showed a correlation with the occupational SARS-CoV-2 infection risk. This online follow-up survey aims to examine changes in mental distress as the pandemic progressed in Germany and to identify factors influencing potential changes.

**Methods:**

Longitudinal data from 260 subjects were available for this analysis. Mental distress related to anxiety and depression symptoms, assessed by the Patient Health Questionnaire-4 (PHQ-4), and occupational risk factors were solicited at the end of 2022 and retrospectively at the fifth wave. Categorized PHQ-4 scores were modelled with mixed ordinal regression models and presented with odds ratios (OR) and 95% confidence intervals (95% CI).

**Results:**

A previous diagnosis of a depressive or anxiety disorder was a strong risk factor for severe symptoms (OR 3.49, 95% CI 1.71–7.11). The impact of occupational SARS-CoV-2 infection risk on mental distress was increased, albeit failing to reach the formal level of statistical significance (high risk OR 1.83, 95% CI 0.59–5.63; probable risk OR 1.72, 95% CI 0.93–3.15). Mental distress was more pronounced in those with a previous diagnosis of anxiety and depression. Confirmed occupational risk factors were protective measures against occupational SARS-CoV-2 infection perceived as inadequate, chronic work-related stress, overcommitment, reduced interactions with fellow-workers, and work-privacy conflicts.

**Conclusions:**

The pandemic had a negative impact on anxiety and depression symptoms among the studied non-healthcare workers, particularly early in the pandemic, although this effect does not appear to be permanent. There are modifiable risk factors that can protect workers’ mental health, including strengthening social interactions among employees and reducing work-privacy conflicts.

## Introduction

The 2019 coronavirus (COVID-19) pandemic is likely the most widespread and sincere crisis in modern occupational medicine. Although the entire working population was affected, the risk of workplace exposure to SARS-CoV-2 varied by occupation and industry and changed over the course of the pandemic. At the beginning of the pandemic, there was an increased risk of contracting SARS-CoV-2 or dying from COVID-19, especially for healthcare professionals [1, 2], but also for employees in social care, transportation, waste collection, safety and security occupations, or agriculture [1–5]. Other workers, such as teachers, cooks, or bartenders, were at greater risk in later pandemic waves when schools and businesses reopened [2, 4].

With the outbreak of the COVID-19 pandemic, various safety measures were introduced to prevent the transmission of the virus in the workplace. Until vaccines became available, these included general recommendations to keep a safe distance, to wear masks, to implement additional hand hygiene, travel bans, closures of educational institutions and non-essential businesses, and contact reduction measures, which notably included the introduction of home-based work [6, 7]. Thus, the COVID-19 pandemic dramatically altered modes of working, and many infection control practices designed to reduce social contacts were associated with isolation, loneliness, and psychological distress [8–10]. However, perceived support at work during the pandemic was associated with a lower risk of anxiety and depression, as shown in a study among healthcare workers [11].

In addition to the fear of contagion, insecure economic conditions, or unemployment had a negative impact on mental health and led to increased symptoms of depression and anxiety among the German general population during the first year of the COVID-19 pandemic [12, 13]. These findings were also evident in systematic reviews conducted in the early days of the pandemic, both in the general population and particularly among healthcare workers [14, 15]. Although healthcare professionals, especially nurses working in COVID-19 wards, are likely to be most affected by the psychosocial consequences of the pandemic [16, 17], negative effects were also observed among other occupational groups. For example, a study among 842 union grocery store workers in California in late 2020 showed that COVID-19-related fear, workplace threat perception, and overall perceived stress affected workers‘ mental health [18]. Increased symptoms of depression and anxiety or higher psychological distress were also observed among social workers, teachers or bank employees during the COVID-19 pandemic [19–21]. In addition, the baseline survey underlying this study during the second and third pandemic waves in Germany (December 2020 to June 2021) with a retrospective survey on mental distress in spring 2020, showed that non-healthcare professionals with high and probable occupational SARS-CoV-2 infection risk (OSIR) were at higher risk for depressive and anxiety symptoms [22].

This follow-up study was conducted to investigate the following research questions among a population of non-healthcare professionals in Germany: (1) How has workers’ mental distress changed during the three years of the COVID-19 pandemic? (2) Can the association between workplace-specific SARS-CoV-2 infection risk and workers’ mental distress observed at the beginning of the pandemic be confirmed at later time points in the pandemic? (3) Which factors influence a potential change in mental distress in later pandemic waves?

## Materials and methods

### Study design and study population

The results presented here are based on the follow-up survey of employees who participated in the baseline survey on mental distress during the COVID-19 pandemic, conducted between 7 December 2020 and 28 June 2021. The rationale, study design, conduct, and results of the baseline survey have been described in detail elsewhere [22]. In brief, all employees of participating companies and facilities, which were recruited by the Social Accident Insurance Institutions for the raw materials and the chemical industry (BG RCI), for the administrative sector (VBG), for the trade and logistics industry (BGHW), and for the public sector in Hesse (Unfallkasse Hessen), were eligible and received a participation link to the baseline online survey via their employers. In the baseline survey, 563 of the 1,545 subjects agreed to be interviewed again and provided their email addresses. An email invitation to the follow-up survey was sent by the study’s trustee on 2 November 2022. As in the baseline survey, participation in the follow-up survey was voluntary. All participants agreed to the privacy policy and provided informed consent online. Both surveys (baseline and follow-up) were approved by the Ethics Committee of the Ruhr University Bochum, Germany (Reg. No. 20–7072). We followed STROBE reporting guidelines for observational studies [23].

By the end of follow-up recruitment on 9 January 2023, 359 completed questionnaires had been collected, of which 20 subjects completed the questionnaire twice. One subject who did not answer questions on mental distress and 63 participants without baseline data were excluded. In addition, 15 employees with a new AD diagnosis during the pandemic were excluded because it could not be ruled out that they had fallen ill as a result of the pandemic. Thus, data from 260 subjects were available for this analysis. The baseline survey assessed mental distress at the first wave (t1, retrospective) and between the peak of the second wave and the end of the third wave (t2). The follow-up included the survey of the fifth wave (t3, retrospective) and the survey at the end of 2022 (t4). S1 File shows recruitment periods as well as 7-day SARS-CoV-2 incidences and pandemic waves in Germany [24].

### Follow-up questionnaire

The follow-up questionnaire used was a shortened version of the baseline questionnaire using the same validated instruments to assess mental and occupational stress as before [22]. The brief 4-item Patient Health Questionnaire (PHQ-4), combining the 2-item PHQ-2 and the 2-item Generalized Anxiety Disorder (GAD)-2 scales, was used to rate mental distress in terms of depression and anxiety symptoms at t3 and t4. PHQ-4 scores were categorized as normal (0–2), mild (3–5), moderate (6–8), and severe (9–12), whereas a score greater than or equal to 3 on the 0-to-6-point PHQ–2 and GAD-2 subscales indicated a probable major depressive disorder or a probable generalized anxiety disorder, respectively [25, 26]. Work-privacy conflicts were surveyed at t3 and t4 analogous to the German version of the Copenhagen Psychosocial Questionnaire (COPSOQ) using the question “To what extent do the demands of your work interfere with your private and family life?” [27]. The middle category (“to some extent”) was used as reference and compared to high and low work-privacy conflicts. Perceived organizational support for infection prevention was evaluated with the following item: “Do you feel protected from SARS-CoV-2 infection by your employer’s policies?”. The response options were ‘yes’, ‘no’, and ‘I do not know’. Contact with colleagues and supervisors was evaluated with the following item: “Have you suffered from reduced contact with your colleagues or your supervisor due to the SARS-CoV-2-related preventive measures (e.g., home office, staggered working hours)?”. The response options were ‘Social interaction was not reduced’, ‘I did not suffer’, ‘I suffered a little’, and ‘I suffered a lot’.

Chronic work-related stress was assessed as imbalance between occupational effort and reward using the short version of the effort-reward imbalance questionnaire, where a ratio of effort to reward above one indicates a high level of effort that is not met by rewards received or expected [28]. Intrinsic effort was assessed as overcommitment to work using the overcommitment questionnaire [29]. Overcommitment was used as a continuous variable, with higher scores being more indicative of excessive engagement. As both instruments are long-term parameters, chronic work-related stress and overcommitment were queried during follow-up only at t4.

Participants were asked about demographics, their general health, and, in contrast to the baseline questionnaire, additionally about previous diagnoses of an anxiety disorder or depression (AD diagnosis) before and during the pandemic. Possible occupational changes since the baseline survey were assessed. Based on participants’ occupational and industry information, we assigned them either a high, probable, or no elevated risk of work-place SARS CoV-2 infection for each study period [22]. Participants with missing or insufficient occupational information were summarized into the group ‘assignment not possible’. Most of the participants in the high-risk group were employees in social work or education, and those in the group at probable SARS-CoV-2 infection risk were employees in the public service or financial sector (S2 File). To circumvent small group sizes, a three-level variable of occupational SARS-CoV-2 infection risk (high and probable; no; assignment not possible) was also analyzed.

### Statistical analysis

Continuous variables were characterized by median and interquartile range (IQR) and categorical variables by number and percent. Scores obtained at different time points were compared using Wilcoxon signed-rank tests. McNemar tests or Bowker tests for symmetry were applied to paired categorical data, i.e., when comparing data from t2 and t4. Group comparisons of continuous variables were performed by Kruskal-Wallis tests (KWT).

Mental distress assessed with the 4-category PHQ-4 variable was modeled with ordinal random-intercept regression models using the SAS procedure PROC GLIMMIX, accounting for multiple measurements per participant (mixed models). Possible factors influencing mental distress were first examined using univariate mixed models and presented with odds ratios (OR) and 95% confidence intervals (95% CI). Adjustment variables for the association between occupational SARS-CoV-2 infection risk (independent variable) and mental distress (dependent variable), which had already been considered in the baseline study, were also used in the multiple regression models of the follow-up data. These included work-privacy conflicts, perceived adequate protection at work, suffering from reduced contact with colleagues, overcommitment to work, sex, age, and time of survey. In addition, the presence of a previous AD diagnosis was also taken into account as an influencing factor on mental distress during the follow-up. Factors influencing major depressive symptoms (PHQ-2 ≥ 3) and major anxiety symptoms (GAD-2 ≥ 3) were estimated with mixed logistic regression models. Univariate regression models were always adjusted by the time of the survey (t1—wave 1 retrospectively; t2—wave 2 and 3; t3—wave 5 retrospectively; t4—end of 2022). For multiple regression models, the estimators of the adjustment variables are reported in the corresponding tables. All statistical analyses were performed using SAS software, version 9.4 (SAS Institute Inc., Cary, NC, USA). Graphs were prepared with GraphPad Prism, version 9 (GraphPad Software, La Jolla, California, USA).

## Results

### Study population

At follow-up, the distribution of SARS-CoV-2 risk groups among the 260 participants was similar to that of the study population at baseline: 6.5% at high risk, 31.5% at probable risk, 54.6% without increased risk, and 7.3% without assignment. However, slightly more women (58.1% *vs*. 52.6%) and statistically significantly more persons with a university degree (50.8% *vs*. 43.6%) participated in the follow-up (t4) compared with the baseline survey (t2). Median age at t4 was 48 years (IQR 38–56). More details on sociodemographic characteristics of the follow-up study population are shown in S3 File.

Table 1 shows the changes in occupational strain in the follow-up study population compared with baseline. Over the course of the pandemic (t2 to t4), participants were more likely to report low work-privacy conflicts (48.5% *vs*. 57.7%, P_McNemar_ = 0.065) and less likely to report suffering from reduced contact with colleagues (62.7% *vs*. 20.4%, P_Bowker_<0. 001). However, although the majority of workers felt adequately protected from a SARS-CoV-2 infection at their workplace at t2 (75.8%) and t3 (81.5%), this proportion reduced to 64.6% at t4 (t2 *vs*. t4, P_McNemar_ = 0.001). Among educational and social work professionals and other employees with high occupational SARS-CoV-2 infection risk, only 41% felt adequately protected at t4. However, from t2 to t4, little increase in the proportion of individuals with chronic work-related stress was observed (58.8% *vs*. 60.4%, P_McNemar_ = 0.541).

**Table 1:** Characterization of occupational strain in the follow-up study population.

		Baseline	Follow-Up		
		t2	t3	t4	t2 vs. t4
		N (%*)	N (%*)	N (%*)	P value
Chronic work-related	Yes (ERI score > 1)	153 (58.8)		157 (60.4)	0.541^a^
stress	No	106 (40.8)		101 (38.8)	
ERI score	Median (IQR)	1.10 (0.88–1.37)		1.17 (0.89–1.51)	0.052^b^
Effort score [3–12]	Median (IQR)	9 (8–10)		9 (8–10.75)	0.827^b^
Reward score [7–28]	Median (IQR)	19 (16.8–21)		18 (15.2–21)	0.110^**b**^
Overcommitment [6–24]	Median (IQR)	15 (13–17)		15 (12–18)	0.052^**b**^
Work-privacy conflicts	High	44 (16.9)	52 (20.0)	37 (14.2)	0.065^**a**^
	Moderate	89 (34.2)	72 (27.7)	72 (27.7)	
	Low	126 (48.5)	135 (51.9)	150 (57.7)	
Perceived adequate protection at work	No / Do not know	57 (21.9)	48 (18.5)	91 (35.0)	**0.001** ^**a**^
	Yes	197 (75.8)	212 (81.5)	168 (64.6)	
Suffered from reduced	Yes	163 (62.7)	125 (48.1)	53 (20.4)	**<0.001** ^a^
contact with colleagues	No	69 (26.5)	99 (38.1)	112 (43.1)	
	Not reduced	28 (10.8)	36 (13.8)	95 (36.5)	

*t2*, waves 2 & 3; *t3*, wave 5; *t4*, end of 2022; *IQR*, interquartile range; *ERI*, effort-reward imbalance

*The percentages do not always add up to 100% because not each participant answered every question

^a^p value of McNemar tests or Bowker tests for symmetry

^b^p value of Wilcoxon signed-rank tests.

### Factors associated with mental distress

**Fig 1:**
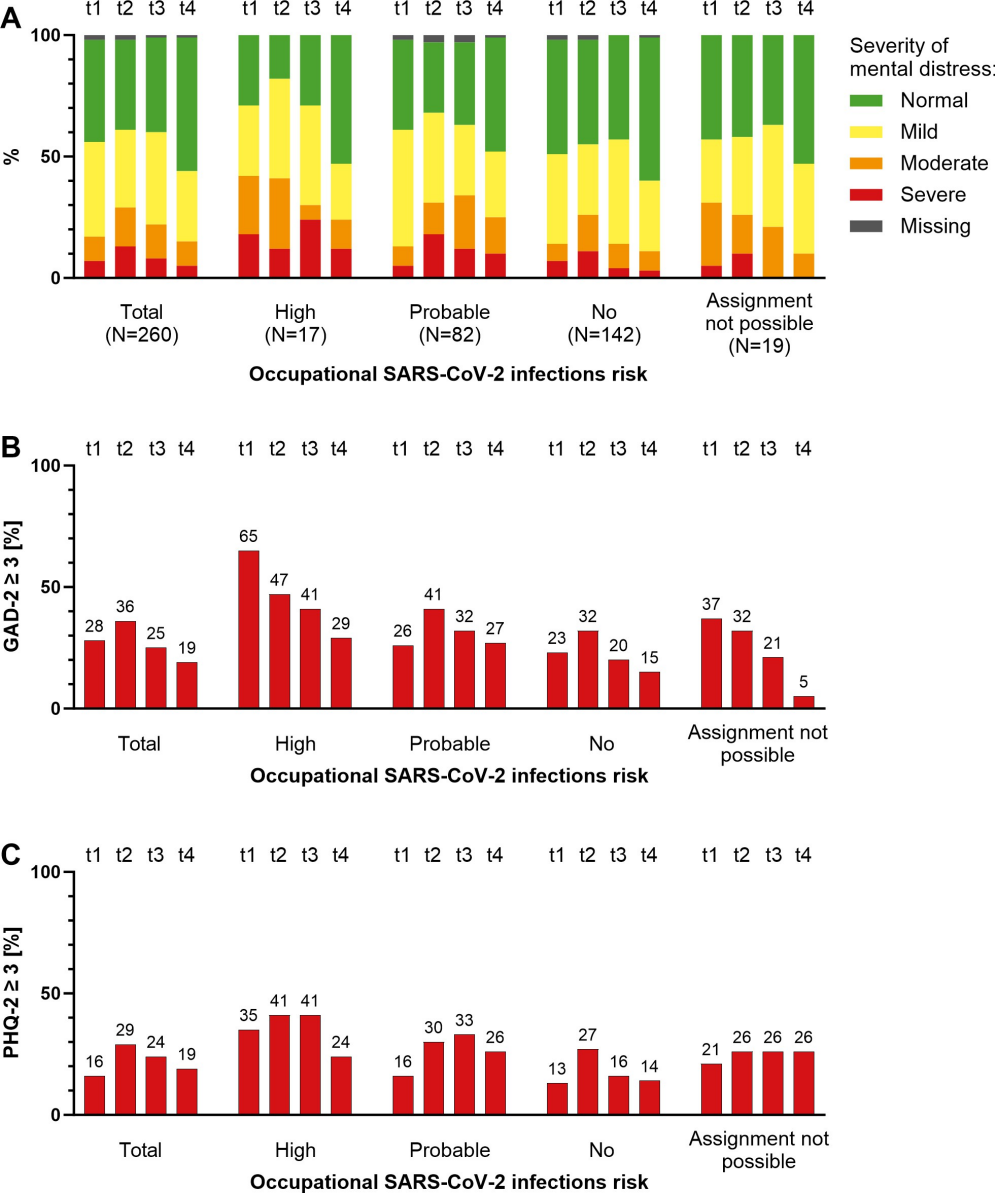
Distribution of mental distress (A), probable generalized anxiety disorder (B), and probable major depressive disorder (C) by occupational SARS-CoV-2 risk group and time of survey for 260 participants with baseline and follow-up data. Mental distress is assessed using the PHQ-4 score indicating the severity of anxiety and depressive symptoms. GAD-2 scores ≥ 3 and PHQ-2 scores ≥ 3 indicate a probable generalized anxiety disorder and a probable major depressive disorder, respectively. The survey was conducted during wave 1 (t1, retrospective), waves 2 and 3 (t2), wave 5 (t3, retrospective), and at the end of 2022 (t4).

The mental distress of the study population changed over the course of the pandemic (Fig 1). Overall, mental distress was highest at t2 with a median PHQ-4 score of 4 (IQR 1–6) and 13% of participants experiencing severe anxiety and depression symptoms (PHQ-4 score ≥ 9). A probable anxiety disorder (GAD-2 ≥ 3) was recorded in 36% of employees and a probable depressive disorder (PHQ-2 ≥ 3) in 29% of employees at t2. Employees with high occupational SARS-CoV-2 infection risk had the highest symptom burden at all time points, with total distress being highest at t3 (24% with PHQ-4 score ≥ 9). Overall, the lowest symptom severity was seen at t4 (PHQ-4 median 2, IQR 0–4).

**Fig 2:**
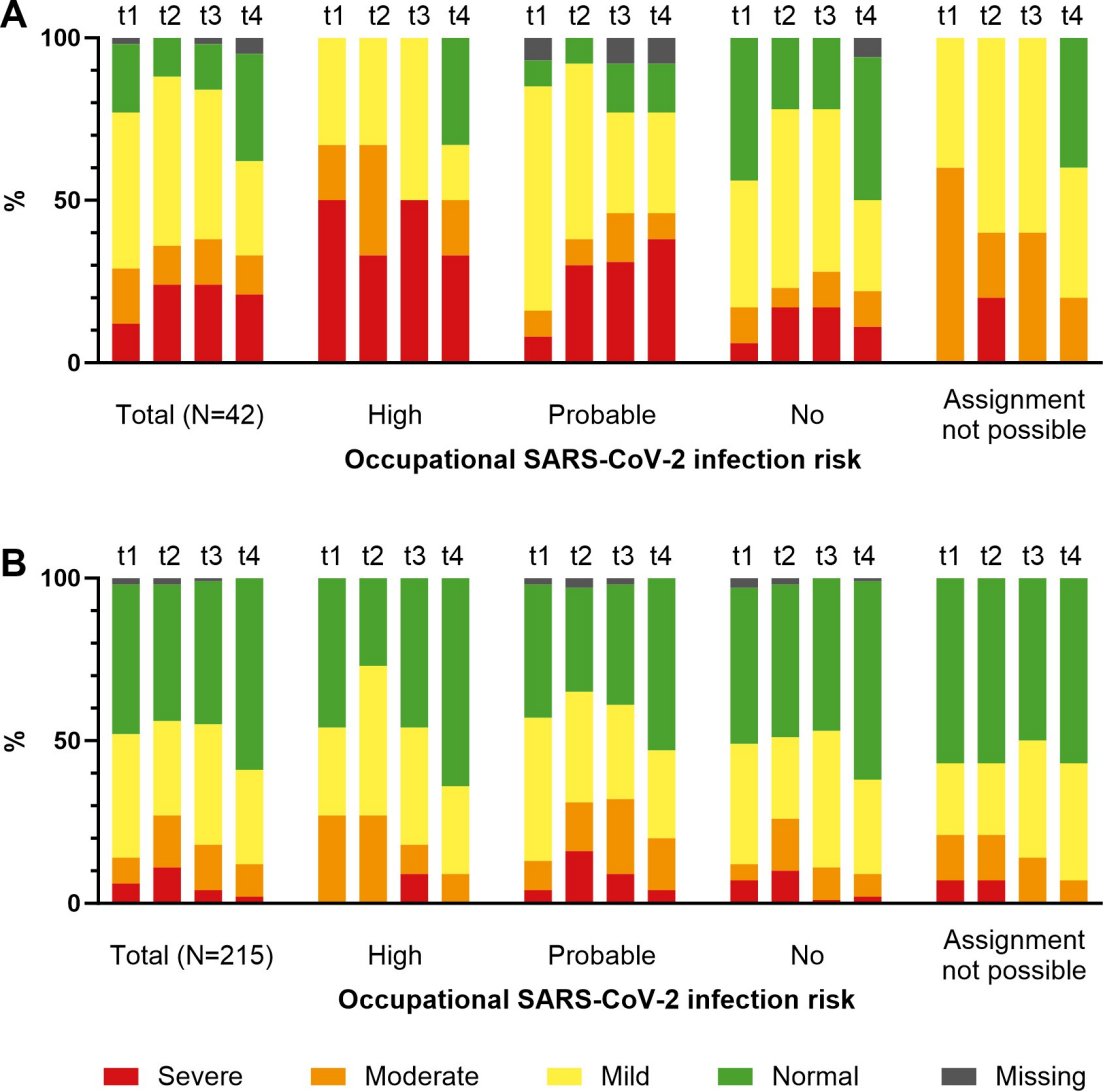
Severity of mental distress of subjects with (A) and without (B) a previous diagnosis of anxiety disorder or depression by occupational SARS-CoV-2 risk group and time of survey. Mental distress is assessed using the PHQ-4 score indicating the severity of anxiety and depressive symptoms as normal (green), mild (yellow), moderate (orange) and severe (red), with missing values shown in gray. The survey was conducted during wave 1 (t1, retrospective), waves 2 and 3 (t2), wave 5 (t3, retrospective), and at the end of 2022 (t4).

At t4, 42 participants (16.2%) reported that they had been diagnosed with an AD diagnosis before the pandemic. Participants with a pre-existing AD diagnosis were more likely to be women (71%) to suffer from chronic work-related stress (79% at t4), have high work-private conflicts (26% at t4), were less likely to feel protected at work (43% at t4), and had higher levels of overcommitment. The severity of anxiety and depression symptoms assessed by the PHQ-4 differed at each time point between individuals with a pre-existing AD diagnosis and without AD diagnosis (Fig 2). PHQ-4 median scores were higher for those with a pre-existing AD diagnosis than those without AD diagnosis (t1: 4 (IQR 3–6) *vs*. 3 (1–4), p_KWT_<0.001; t2: 5 (4–8) *vs*. 3 (1–6), p_KWT_ = 0.001; t3: 4 (3–8) *vs*. 3 (1–5), p_KWT_<0.001; t4: 4 (1.5–8) *vs*. 2 (0–4), p_KWT_<0.001).

Employees’ mental distress was influenced by many different factors, including increased work-related SARS-CoV-2 infection risk (high risk: OR 3.76, 95% CI 1.11–12.76; probable risk: OR 2.36, 95% CI 1.23–4.54) and, in particular, by a pre-existing AD diagnosis (Table A in S4 File). The risk for depressive and anxiety symptoms was increased fivefold among employees with AD diagnosis before the pandemic, compared with employees without AD diagnosis. Increased mental distress was also evident for women (OR 2.73; 95% CI 1.51–4.93), single parents, employees with high work-privacy conflicts, with higher overcommitment to work, with chronic work-related stress, perceiving inadequate protection at work, with reduced contact with colleagues, and with less good general health. Univariate models stratified by AD diagnosis revealed that, particularly among employees with AD diagnosis, high occupational SARS-CoV-2 infection risk led to increased risks of more severe symptoms (Table B in S4 File). In contrast, the risk for more severe symptoms was increased in individuals without AD diagnosis among employees with probable occupational SARS-CoV-2 infection risk (OR 2.19, 95% CI 1.11–4.33), among women compared with men (OR 2.35, 95% CI 1.28–4.32), and among employees who suffered from reduced contact with colleagues (OR 5.32, 95% CI 2.67–10.58) or who perceived inadequate protection from workplace infection (OR 1.95, 95% CI 1.22–3.12). Work-privacy conflicts and overcommitment to work were risk factors for more severe symptoms for all subjects.

The multiple mixed models adjusted estimates are shown for all employees in Table 2 and stratified by AD diagnosis in Table C in S4 File. In the total study population, the effect of high occupational SARS-CoV-2 infection risk on more severe symptoms was reduced (OR 1.83, 95% CI 0.59–5.63), and the effect of probable occupational SARS-CoV-2 infection risk was slightly reduced (OR 1.72, 95% CI 0.93–3.15) compared to the univariate analysis. The strongest risk factors for more severe symptoms were suffering from reduced contact with colleagues and a pre-existing AD diagnosis. The analysis with a three-level variable of occupational SARS-CoV-2 infection risk (high and probable; no; assignment not possible) also showed that the influence of the occupational SARS-CoV-2 infection risk on mental distress was particularly evident in the group of employees with AD diagnosis (Table D in S4 File).

**Table 2:** Multiple regression model of the association between mental distress and occupational SARS-CoV-2 infection risk.

		OR	95% CI	
Occupational SARS-CoV-2	High	1.83	0.59	5.63
infection risk	Probable	1.72	0.93	3.15
	Assignment not possible	1.16	0.39	3.45
	None (ref)	1.00		
AD diagnosis	Before pandemic	**3.49**	**1.71**	**7.11**
	Never (ref)	1.00		
Work-privacy conflicts	High	**1.77**	**1.00**	**3.12**
	Moderate (ref)	1.00		
	Low	0.60	0.36	1.00
Perceived adequate protection	No/Do not know	**1.79**	**1.15**	**2.76**
	Yes (ref)	1.00		
Suffered from reduced contact with colleagues	Yes	**4.16**	**2.27**	**7.64**
	No	**1.93**	**1.07**	**3.46**
	Not reduced (ref)	1.00		
Overcommitment to work [6–24]		**1.22**	**1.13**	**1.32**
Sex	Women	**2.15**	**1.24**	**3.75**
	Men (ref)	1.00		
Age (per 10 years)		0.69	0.52	0.92
Time of survey	t4	0.85	0.51	1.41
	t3	1.45	0.94	2.25
	t2	**2.10**	**1.42**	**3.12**
	t1 (ref)	1.00		

Mental distress assessed with the four-category PHQ-4 variable was modelled with a multiple ordinal random-intercept regression model.

*OR*, odds ratio; *CI*, confidence interval; *ref*, reference; *AD diagnosis*, diagnosed anxiety disorder or depression; *t4*, end of 2022; *t3*, wave 5 (retrospective); *t2*, waves 2 & 3; *t1*, wave 1 (retrospective)

### Differential effects on anxiety and depressive symptoms

Separate analyses for anxiety and depressive symptoms did not show a statistically significant effect of high and probable occupational SARS-CoV-2 infection risk on the risk of severe anxiety symptoms (GAD-2 ≥ 3, Table 3) or severe depressive symptoms (PHQ-2 ≥ 3, Table 4) when all participants were considered. In contrast, a pre-existing AD diagnosis, female sex, overcommitment to work, perceived inadequate protection against workplace infections, and suffering from reduced contact with colleagues were risk factors for the occurrence of both, severe anxiety symptoms and severe depressive symptoms. In addition, severe anxiety symptoms were more likely than depressive symptoms to be influenced by high work-private conflicts. The strongest risk factor for severe depressive symptoms was suffering from reduced contact with colleagues (OR 3.92, 95% CI 1.87–8.23). Stratified analysis showed that among workers diagnosed with AD, a high occupational risk of SARS-CoV-2 infection was more likely to result in an increased risk of severe anxiety symptoms (OR 7.87, 95% CI 1.83–33.8) than severe depression symptoms (OR 5.43, 95% CI 0.35–83.4).

**Table 3:** Risk estimation for severe anxiety symptoms (GAD-2 ≥ 3).

		Total			Pre-existing AD diagnosis			Never AD diagnosis		
		OR	95% CI		OR	95% CI		OR	95% CI	
Occupational	High	1.85	0.74	4.65	**7.87**	**1.83**	**33.76**	0.99	0.29	3.35
SARS-CoV-2	Probable	1.15	0.67	1.99	2.82	0.78	10.15	1.05	0.57	1.93
infection risk	Assignment not possible	0.89	0.39	2.04	2.06	0.35	12.05	0.60	0.23	1.59
	None (ref)	1.00			1.00			1.00		
AD diagnosis	Before pandemic	**2.23**	**1.18**	**4.20**						
	Never	1.00								
Work-privacy	High	**2.21**	**1.17**	**4.17**	2.53	0.72	8.85	**2.31**	**1.07**	**4.99**
conflicts	Moderate (ref)	1.00			1.00			1.00		
	Low	0.75	0.42	1.32	1.05	0.26	4.27	0.81	0.43	1.53
Perceived	No/Do not know	1.56	0.99	2.44	1.49	0.54	4.11	1.56	0.91	2.66
adequate protection	Yes (ref)	1.00			1.00			1.00		
Suffered from	Yes	**2.39**	**1.22**	**4.68**	0.50	0.11	2.28	**3.52**	**1.59**	**7.81**
reduced contact	No	0.88	0.48	1.63	0.56	0.16	2.02	1.07	0.50	2.29
with colleagues	Not reduced (ref)	1.00			1.00			1.00		
Overcommitment to work [6–24]		**1.21**	**1.11**	**1.31**	**1.28**	**1.04**	**1.56**	**1.20**	**1.09**	**1.32**
Sex	Women	**2.83**	**1.63**	**4.89**	1.25	0.34	4.52	**3.27**	**1.82**	**5.89**
	Men (ref)	1.00			1.00			1.00		
Age (per 10 years)		0.89	0.69	1.14	0.87	0.44	1.71	0.87	0.66	1.14
Time of survey	t4	0.67	0.35	1.28	0.35	0.09	1.32	0.78	0.36	1.68
	t3	0.82	0.46	1.44	0.78	0.18	3.38	0.81	0.43	1.55
	t2	**1.70**	**1.05**	**2.74**	1.75	0.49	6.26	1.65	0.96	2.85
	t1 (ref)	1.00			1.00			1.00		

Severe anxiety symptoms were modelled with multiple mixed logistic regression models.

*GAD-2*, 2-item Generalized Anxiety Disorder scale, *OR*, odds ratio; *CI*, confidence interval; *ref*, reference; *AD diagnosis*, diagnosed anxiety disorder or depression; *t4*, end of 2022; *t3*, wave 5 (retrospective); *t2*, waves 2 & 3; *t1*, wave 1 (retrospective)

**Table 4:** Risk estimation for severe depressive symptoms (PHQ-2 ≥ 3).

		Total			Pre-existing AD diagnosis			Never AD diagnosis		
		OR	95% CI		OR	95% CI		OR	95% CI	
Occupational	High	1.88	0.64	5.54	5.43	0.35	83.39	1.02	0.31	3.32
SARS-CoV-2	Probable	1.40	0.69	2.85	2.62	0.25	27.37	1.50	0.69	3.26
infection risk	Assignment not possible	1.84	0.47	7.15	3.01	0.07	125.44	1.60	0.37	6.97
	None (ref)	1.00			1.00			1.00		
AD diagnosis	Before pandemic	**2.47**	**1.10**	**5.57**						
	Never	1.00								
Work-privacy	High	1.39	0.70	2.76	1.09	0.15	7.75	1.63	0.77	3.44
conflicts	Moderate (ref)	1.00			1.00			1.00		
	Low	0.45	0.23	0.88	0.23	0.03	1.91	0.58	0.28	1.18
Perceived	No/Do not know	**1.81**	**1.09**	**3.01**	2.75	0.65	11.57	1.56	0.85	2.87
adequate protection	Yes (ref)	1.00			1.00			1.00		
Suffered from	Yes	**3.92**	**1.87**	**8.23**	0.71	0.12	4.14	**7.92**	**2.99**	**20.98**
reduced contact	No	1.77	0.81	3.85	0.62	0.13	2.91	**2.96**	**1.06**	**8.28**
with colleagues	Not reduced (ref)	1.00			1.00			1.00		
Overcommitment to work [6–24]		**1.22**	**1.12**	**1.33**	**1.38**	**1.00**	**1.90**	**1.22**	**1.10**	**1.34**
Sex	Women	**2.18**	**1.14**	**4.20**	0.40	0.05	3.16	**3.21**	**1.55**	**6.62**
	Men (ref)	1.00			1.00			1.00		
Age (per 10 years)		**0.65**	**0.48**	**0.89**	0.67	0.19	2.41	**0.63**	**0.45**	**0.88**
Time of survey	t4	**2.09**	**1.01**	**4.35**	1.15	0.17	7.96	**2.57**	**1.07**	**6.18**
	t3	**2.19**	**1.12**	**4.27**	1.27	0.26	6.17	**2.58**	**1.15**	**5.80**
	t2	**3.23**	**1.75**	**5.96**	1.98	0.31	12.85	**3.71**	**1.84**	**7.47**
	t1 (ref)	1.00			1.00			1.00		

Severe depressive symptoms were modelled with multiple mixed logistic regression models.

*PHQ-2*, 2-item Patient Health Questionnaire, *OR*, odds ratio; *CI*, confidence interval; *ref*, reference; *AD diagnosis*, diagnosed anxiety disorder or depression; *t4*, end of 2022; *t3*, wave 5 (retrospective); *t2*, waves 2 & 3; *t1*, wave 1 (retrospective)

## Discussion

This study highlights the major importance of occupational factors on mental health during the COVID-19 pandemic among non-healthcare employees in Germany. The pandemic was associated with increased mental distress among the surveyed employees with overall highest symptom severity at t2, whereas educational and social work professionals and employees in other jobs at high occupational SARS-CoV-2 infection risk were particularly affected at t3. The observed increase of anxiety and depression symptoms appears to be transient during the pandemic, as symptom severity regressed by t4. A previous AD diagnosis was a strong risk factor for more severe anxiety and depression symptoms during the pandemic. Among employees without a pre-existing diagnosis, the impact of occupational SARS-CoV-2 infection risk on mental distress decreases with decreasing symptom severity during the pandemic. Other occupational risk factors for mental distress that were found in the baseline study and remained in the follow-up were overcommitment to work, chronic work-related stress, perceived inadequate protection against SARS-CoV-2 at work, reduced contact with colleagues, and work-privacy conflicts.

This analysis had the strength of using follow-up data from a large survey of employees from different occupational sectors other than the health sector with different occupational SARS-CoV-2 infection risks. Validated scales were used to assess depression and anxiety symptoms, chronic work-related stress, and overcommitment to work among others at up to four time points during the pandemic. However, this follow-up study also has limitations. Limitations include retrospective data collection at t1 and t3. Therefore, this study may be subject to well-known biases of retrospective studies, such as recall and selection bias. Second, despite the large study population at baseline, data from only 260 subjects could be analyzed during follow-up. Hence, statistical power was limited, especially in the stratified analysis. Third, participation in the baseline and follow-up surveys was voluntary, so only interested individuals with potentially unique characteristics participated, making it difficult to generalize the results to all employees (e.g., 51% of participants had a university degree, twice as many as in the general German population).

We demonstrated that depression and anxiety symptoms were common among the studied employees in Germany and differed notably between occupational SARS-CoV-2 infection risk groups and time of interview. In our study, prevalence proportions of probable depression and anxiety were remarkably higher at t2 (depressive symptoms: 29% *vs*. 24%, anxiety symptoms: 36% *vs*. 22%), but similar at t3 (depressive symptoms: 24% *vs*. 24%, anxiety symptoms: 25% *vs*. 24%) compared to another study [30, 31]. Higher but also lower prevalence proportions during the pandemic were reported in other studies from Germany [13, 32, 33] or in a recent umbrella review [34]. Heterogeneity in prevalence proportions could be due to differences in survey design, survey focus, survey period, study location, or assessment instruments and cutoffs.

Including the 15 subjects who received an AD diagnosis during the pandemic, 57 individuals (20.7%) reported at t4 that they had been diagnosed with an anxiety disorder or depression. This is in the same order of magnitude as the sum of recorded anxiety disorders (15%) and major depressive disorders (6%) from a general population sample conducted during the first months of the COVID-19 pandemic in Germany [35]. Consistent with other studies, in our study participants with pre-existing mental health disorders were more likely to report related symptoms than individuals without an AD diagnosis [35, 36].

In addition, within the follow-up study, as in the baseline survey, we were able to confirm known risk factors for mental distress, such as female sex, the presence of work-privacy conflicts, or perceived work-related stress [37–39]. Consistent with observations from a representative sample from Europe [30], but in contrast to a European review [39], we did not find an association between educational status and symptom severity.

Another influencing factor during the pandemic that negatively affected mental health was reduced contact with colleagues. This is in line with observations among U.S. employees in the initial phase of the pandemic which linked work loneliness to more depressive symptoms [10]. A nationwide online cross-sectional survey in Japan among office workers conducted in December 2020 found further that low levels of co-worker or supervisor support are strongly associated with loneliness [40]. Hence, co-worker and supervisor support could be a critical factor in preventing loneliness (for example due to remote work) and associated mental health problems. In addition, the implementation of appropriate infection control practices and interventions in the workplace can also have a positive impact on employees’ mental health, as these measures can make employees feel safer in their workplace [22, 41, 42]. Perceptions of an unsafe workplace environment, low levels of institutional trust, and a lack of control are known mental health stressors and have been observed in several occupations during the COVID-19 pandemic, such as supermarket workers [43], teachers [44], or health care workers [45]. The negative impact of perceived inadequate protection from SARS-CoV-2 in the workplace on anxiety and depressive symptoms was also confirmed in this survey.

Consistent with two large longitudinal cohorts from the UK that found anxiety to be the most affected aspect of mental health during the pandemic [36], we also observed more pronounced anxiety symptoms than depression symptoms. The effects of work-privacy conflicts were stronger for anxiety symptoms. The influence of occupational SARS-CoV-2 infection risk on elevated GAD-2 scores was statistically significant after adjustment in the group of employees with AD diagnosis. On the other hand, work loneliness (assessed as suffering from reduced contact with colleagues) and perceived inadequate protection against SARS-CoV-2 infections in the workplace had a stronger effect on depressive symptoms than on anxiety symptoms in our study population of non-healthcare employees.

## Conclusions

During the COVID-19 pandemic, work-related psychosocial risks played a crucial role in the well-being of the non-healthcare employees surveyed. Although the effects of the pandemic on anxiety and depressive symptoms appeared to be temporary, modifiable factors to protect the mental health of all employees remain after the pandemic ended, namely reduced contact with fellow-workers and work-privacy conflicts. Hence, employers should create opportunities for social interaction among employees to minimize workplace loneliness and provide appropriate support for employees with work-privacy conflicts or a known AD diagnosis.

## Supporting information

S1 File Survey periods of the study in relation to SARS-CoV-2 incidences and pandemic waves with the respective prevailing variance of concern (VOC) in Germany. (DOCX)

S2 File Distribution of occupations at follow-up with assigned increased risk of SARS-CoV-2 infection. (DOCX)

S3 File Sociodemographic characteristics of the follow-up study population. (DOCX)

S4 File Additional linear mixed model analysis for mental distress. (DOCX)
