# Supplementary material for: Changes in mental distress among employees during the three years of the COVID-19 pandemic in Germany
Source: PLoS One. 2024 May 3;19(5):e0302020. doi: 10.1371/journal.pone.0302020 (PMC11068204; doi:10.1371/journal.pone.0302020)
Supplement: S1 File — (DOCX) [file pone.0302020.s001.docx]

**S1 File.** Survey periods of the study in relation to SARS-CoV-2 incidences and pandemic waves with the respective prevailing variance of concern (VOC) in Germany
