# Supplementary material for: Changes in mental distress among employees during the three years of the COVID-19 pandemic in Germany
Source: PLoS One. 2024 May 3;19(5):e0302020. doi: 10.1371/journal.pone.0302020 (PMC11068204; doi:10.1371/journal.pone.0302020)
Supplement: S2 File — (DOCX) [file pone.0302020.s002.docx]

**S2 File.** Distribution of occupations at follow-up with assigned increased risk of SARS-CoV-2 infection

| Occupational SARS-CoV-2 infection risk | Occupation | N |
| --- | --- | --- |
| High (N=17) | Social Worker | 8 |
|  | Educators and pedagogues | 5 |
|  | Official guardian | 2 |
|  | Firefighter | 1 |
|  | Retail trade employee | 1 |
| Probable (N=82) | Public administrative staff | 54 |
|  | Bank clerks | 17 |
|  | Occupational safety and health professionals and supervisors | 9 |
|  | Construction and transport equipment operators | 2 |
