# Supplementary material for: Changes in mental distress among employees during the three years of the COVID-19 pandemic in Germany
Source: PLoS One. 2024 May 3;19(5):e0302020. doi: 10.1371/journal.pone.0302020 (PMC11068204; doi:10.1371/journal.pone.0302020)
Supplement: S3 File — (DOCX) [file pone.0302020.s003.docx]

**S3 File.** Sociodemographic characteristics of the follow-up study population

| Characteristics | | N (%*) | |
| --- | --- | --- | --- |
| Age [years] | Median (interquartile range) | 48 (38-56) |  |
| Sex | Female | 151 (58.1) |  |
|  | Male | 106 (40.8) |  |
|  | Missing | 3 (1.2) |  |
| Education | ≤ 10 years of schooling | 57 (21.9) |  |
|  | > 10 years of schooling | 71 (27.3) |  |
|  | University degree | 132 (50.8) |  |
| Sector | Public sector | 120 (46.2) |  |
|  | Industrial enterprises | 89 (34.2) |  |
|  | Financial sector | 27 (10.4) |  |
|  | Local public transport | 4 (1.5) |  |
|  | Others | 20 (7.7) |  |
| Occupational SARS-CoV-2 infection risk | High | 17 (6.5) |  |
|  | Potential | 82 (31.5) |  |
|  | None | 142 (54.6) |  |
|  | Assignment not possible | 19 (7.3) |  |
