# Supplementary material for: Changes in mental distress among employees during the three years of the COVID-19 pandemic in Germany
Source: PLoS One. 2024 May 3;19(5):e0302020. doi: 10.1371/journal.pone.0302020 (PMC11068204; doi:10.1371/journal.pone.0302020)
Supplement: S4 File — (DOCX) [file pone.0302020.s004.docx]

**S4 File.** **Additional linear mixed model analysis for mental distress**

**Table A.** Risk estimation of **mental distress** examined with univariate mixed models

|  |  | **N** | **OR** | **95% CI** | |
| --- | --- | --- | --- | --- | --- |
| Occupational SARS-CoV-2 | High | 17 | **3.76** | **1.11** | **12.76** |
| infection risk | Probable | 82 | **2.36** | **1.23** | **4.54** |
|  | Assignment not possible | 19 | 1.33 | 0.43 | 4.09 |
|  | None (ref) | 142 | 1.00 |  |  |
| AD diagnosis | Before pandemic | 42 | **5.49** | **2.60** | **11.60** |
|  | Never (ref) | 215 | 1.00 |  |  |
| General health | Less good | 71 | **6.25** | **3.85** | **10.16** |
|  | Good (ref) | 93 | 1.00 |  |  |
|  | Very good | 96 | **0.29** | **0.18** | **0.46** |
| Age (per 10 years) |  | 250 | 0.87 | 0.65 | 1.18 |
| Sex | Women | 151 | **2.73** | **1.51** | **4.93** |
|  | Men (ref) | 106 | 1.00 |  |  |
| Single parents | Yes | 6 | **3.06** | **1.19** | **7.88** |
|  | No (ref) | 254 | 1.00 |  |  |
| Work-privacy conflicts | High | 37 | **2.27** | **1.31** | **3.92** |
|  | Moderate (ref) | 71 | 1.00 |  |  |
|  | Low | 152 | 0.43 | 0.27 | 0.68 |
| Perceived adequate protection | No/Do not know | 91 | **2.05** | **1.34** | **3.14** |
|  | Yes (ref) | 169 | 1.00 |  |  |
| Suffered from reduced contact | Yes | 53 | **3.75** | **2.06** | **6.82** |
| with colleagues | No | 112 | 1.56 | 0.89 | 2.71 |
|  | Not reduced (ref) | 95 | 1.00 |  |  |
| Overcommitment to work [6-24] |  | 260 | **1.30** | **1.21** | **1.39** |
| Chronic work-related stress | Yes (ERI score > 1) | 158 | **2.68** | **1.75** | **4.10** |
|  | No (ref) | 101 | 1.00 |  |  |

Mental distress was measured with the four-category PHQ-4 variable. Each risk factor was modeled in a single model adjusted by time of survey.

*N*, subjects with corresponding characteristic at the last time of the survey; *OR,* odds ratio; *CI,* confidence interval; *ref*, reference; *AD diagnosis*, diagnosed anxiety disorder or depression; *ERI*, effort-reward imbalance

**Table B.** Univariate regression models of **mental distress** stratified by anxiety disorder or depression diagnosis status

|  |  | **Pre-existing AD diagnosis** | | | | **Never AD diagnosis** | | | |
| --- | --- | --- | --- | --- | --- | --- | --- | --- | --- |
|  |  | **N** | **OR** | **95% CI** | | **N** | **OR** | **95% CI** | |
| Occupational | High | 6 | **13.61** | **1.71** | **108.06** | 11 | 1.43 | 0.38 | 5.45 |
| SARS-CoV-2 | Probable | 13 | 4.71 | 0.70 | 31.50 | 68 | **2.19** | **1.11** | **4.33** |
| infection risk | Assignment not possible | 5 | 3.61 | 0.57 | 22.91 | 14 | 0.81 | 0.21 | 3.11 |
|  | None (ref) | 18 | 1.00 |  |  | 122 | 1.00 |  |  |
| General health | Less good | 17 | **4.82** | **1.58** | **14.66** | 53 | **6.32** | **3.63** | **11.01** |
|  | Good (ref) | 18 | 1.00 |  |  | 75 | 1.00 |  |  |
|  | Very good | 7 | 0.32 | 0.09 | 1.17 | 87 | **0.30** | **0.18** | **0.49** |
| Age (per 10 years) |  | 41 | 0.90 | 0.30 | 2.67 | 206 | 0.80 | 0.59 | 1.09 |
| Sex | Women | 30 | 2.06 | 0.29 | 14.65 | 118 | **2.35** | **1.28** | **4.32** |
|  | Men (ref) | 11 | 1.00 |  |  | 95 | 1.00 |  |  |
| Single parents | Yes | 1 | - |  |  | 5 | 2.74 | 0.90 | 8.32 |
|  | No (ref) | 41 | - |  |  | 210 | 1.00 |  |  |
| Work-privacy | High | 11 | 2.25 | 0.63 | 8.01 | 24 | **2.19** | **1.17** | **4.08** |
| conflicts | Moderate (ref) | 11 | 1.00 |  |  | 59 | 1.00 |  |  |
|  | Low | 20 | **0.19** | **0.05** | **0.69** | 132 | **0.51** | **0.31** | **0.84** |
| Overcommitment to work |  | 42 | **1.41** | **1.19** | **1.67** | 215 | **1.26** | **1.17** | **1.36** |
| Chronic work- | Yes (ERI > 1) | 33 | 2.19 | 0.74 | 6.47 | 122 | **2.51** | **1.57** | **4.01** |
| related stress | No (ref) | 9 | 1.00 |  |  | 92 | 1.00 |  |  |
| Suffered from | Yes | 12 | 1.45 | 0.35 | 5.95 | 40 | **5.32** | **2.67** | **10.58** |
| reduced contact | No | 12 | 0.64 | 0.17 | 2.43 | 98 | **2.32** | **1.22** | **4.43** |
| with colleagues | Not reduced (ref) | 18 | 1.00 |  |  | 77 | 1.00 |  |  |
| Perceived | No/Do not know | 24 | 1.86 | 0.56 | 6.15 | 64 | **1.95** | **1.22** | **3.12** |
| adequate protection | Yes (ref) | 18 | 1.00 |  |  | 151 | 1.00 |  |  |

Mental distress was measured with the four-category PHQ-4 variable. Each risk factor was modeled in a single model and adjusted by time of survey.

*AD diagnosis*, diagnosed anxiety disorder or depression; *N*, subjects with corresponding characteristic at the last time of the survey; *OR,* odds ratio; *CI,* confidence interval; *ref*, reference; *ERI*, effort-reward imbalance score

**Table C.** Multiple regression models of **mental distress** stratified by anxiety disorder or depression diagnosis status

|  |  | **Pre-existing AD diagnosis (N=40)** | | | **Never AD diagnosis (N=204)** | | |
| --- | --- | --- | --- | --- | --- | --- | --- |
|  |  | **OR** | **95% CI** | | **OR** | **95% CI** | |
| Occupational | High | 7.60 | 0.57 | 101.89 | 1.00 | 0.26 | 3.80 |
| SARS-CoV-2 | Probable | 3.22 | 0.38 | 27.68 | 1.64 | 0.85 | 3.19 |
| infection risk | Assignment not possible | 4.36 | 0.25 | 75.53 | 0.87 | 0.24 | 3.16 |
|  | None (ref) | 1.00 |  |  | 1.00 |  |  |
| Work-privacy conflicts | High | 1.75 | 0.42 | 7.36 | 1.74 | 0.89 | 3.42 |
|  | Moderate (ref) | 1.00 |  |  | 1.00 |  |  |
|  | Low | 0.28 | 0.04 | 1.79 | 0.71 | 0.41 | 1.23 |
| Perceived adequate | No/Do not know | 2.09 | 0.46 | 9.49 | **1.68** | **1.04** | **2.71** |
| protection | Yes (ref) | 1.00 |  |  | 1.00 |  |  |
| Suffered from reduced contact | Yes | 1.89 | 0.33 | 10.79 | **5.84** | **2.88** | **11.82** |
| with colleagues | No | 0.68 | 0.14 | 3.25 | **2.67** | **1.34** | **5.30** |
|  | Not reduced (ref) | 1.00 |  |  | 1.00 |  |  |
| Overcommitment to work | | **1.32** | **1.05** | **1.65** | **1.21** | **1.11** | **1.32** |
| Sex | Women | 1.98 | 0.25 | 15.94 | **2.27** | **1.26** | **4.10** |
|  | Men (ref) | 1.00 |  |  | 1.00 |  |  |
| Age (per 10 years) |  | 0.62 | 0.18 | 2.09 | **0.68** | **0.51** | **0.92** |
| Time of survey | t4 | 0.93 | 0.21 | 4.14 | 0.85 | 0.48 | 1.52 |
|  | t3 | 1.93 | 0.48 | 7.73 | 1.38 | 0.84 | 2.26 |
|  | t2 | 2.42 | 0.66 | 8.80 | **2.08** | **1.35** | **3.21** |
|  | t1 (ref) | 1.00 |  |  | 1.00 |  |  |

Mental distress assessed with the four-category PHQ-4 variable was modelled with multiple ordinal random-intercept regression models, so adjusted odds ratios (OR) and 95% confidence intervals (CI) are presented.

*AD diagnosis*, diagnosed anxiety disorder or depression; *ref*, reference; *t4*, end of 2022; *t3*, wave 5 (retrospective); *t2*, wave 2 & 3; *t1*, wave 1 (retrospective)

**Table D.** Risk estimates for mental disorders as a function of the three-level variable of occupational SARS-CoV-2 infection risk (OSIR) in the total study population and stratified by anxiety and depression (AD) diagnosis

|  |  | **Total** | | | **Pre-existing AD diagnosis** | | | | | **Never AD diagnosis** | | | | |
| --- | --- | --- | --- | --- | --- | --- | --- | --- | --- | --- | --- | --- | --- | --- |
|  |  | **OR** | **95% CI** | | **OR** | **95% CI** | | | **OR** | | **95% CI** | | |  |
| Univariate models |  |  |  |  |  |  |  |  | | |  |  |  |  |
| OSIR | Increased | **2.56** | **1.38** | **4.75** | **6.60** | **1.23** | **35.33** | **2.07** | | | **1.08** | **3.94** |  |  |
|  | Assignment not possible | 1.33 | 0.43 | 4.08 | 3.61 | 0.58 | 22.39 | 0.81 | | | 0.21 | 3.10 |  |  |
|  | None (ref) | 1.00 |  |  | 1.00 |  |  | 1.00 | | |  |  |  |  |
| Multiple models |  |  |  |  |  |  |  |  | | |  |  |  |  |
| OSIR | Increased | 1.74 | 0.97 | 3.10 | 4.33 | 0.62 | 30.52 | 1.53 | | | 0.81 | 2.88 |  |  |
|  | Assignment not possible | 1.16 | 0.39 | 3.44 | 4.32 | 0.25 | 75.50 | 0.87 | | | 0.24 | 3.15 |  |  |
|  | None (ref) | 1.00 |  |  | 1.00 |  |  | 1.00 | | |  |  |  |  |

Odds ratios (OR) and 95% confidence intervals (95% CI) were assessed with ordinal random-intercept regression models accounting for multiple measurements per participant. Estimates from univariate models were only adjusted by time of survey. Multiple models show adjusted estimates analogous to the other multiple models according to age, time of survey, sex, work-privacy conflicts, perceived protection at the workplace, contact with colleagues, overcommitment to work, and if appropriate AD diagnosis.
